# Is Secretory Activation Delayed in Women with Type Two Diabetes? A Pilot Study

**DOI:** 10.3390/nu14071323

**Published:** 2022-03-22

**Authors:** Fiona L. Britten, Ching T. Lai, Donna T. Geddes, Leonie K. Callaway, Emma L. Duncan

**Affiliations:** 1 Department of Obstetric Medicine, Women’s and Newborn Services, Royal Brisbane and Women’s Hospital, Brisbane, QLD 4029, Australia; leonie.callaway@health.qld.gov.au; 2 Faculty of Medicine, University of Queensland, Brisbane, QLD 4006, Australia; 3 School of Molecular Sciences, The University of Western Australia, Crawley, WA 6009, Australia; ching-tat.lai@uwa.edu.au (C.T.L.); donna.geddes@uwa.edu.au (D.T.G.); 4 Department of Twin Research & Genetic Epidemiology, School of Life Course Sciences, Faculty of Life Sciences and Medicine, King’s College London, London SE1 7EH, UK; emma.duncan@kcl.ac.uk; 5 Department of Endocrinology, Guy’s and St Thomas’ NHS Foundation Trust, London SE1 7EH, UK

**Keywords:** Type 2 diabetes, lactation, breastfeeding, secretory activation, lactogenesis, neonatal hypoglycaemia, insulin resistance, obesity

## Abstract

(1) Background: Breastfeeding duration may be reduced in women with type 2 diabetes. Delayed secretory activation (SA) is associated with poorer breastfeeding outcomes; however, no prior studies have examined SA in women with type 2 diabetes. This pilot study aimed to assess SA in women with type 2 diabetes by assessing breastmilk constituents. Secondary aims were to assess breastfeeding rates postpartum, and contributory factors. (2) Methods: A prospective cohort of pregnant women with type 2 diabetes (*n* = 18) and two control groups with age- and parity-matched nondiabetic pregnant women (body mass index (BMI)) matched (*n* = 18) or normal-range BMI (*n* = 18)) were recruited. Breastmilk constituents (citrate, lactose, protein, and fat) were measured twice daily for 5 days postpartum and compared between groups. Associations between peripartum variables, breastmilk constituents, and breastfeeding at 4 months postpartum were explored. (3) Results: Women with type 2 diabetes had a slower increase in breastmilk citrate concentration postpartum, indicative of delayed SA, compared to both control groups. Higher predelivery insulin doses in women with type 2 diabetes were associated with increasing time to SA. Both women with type 2 diabetes and BMI-matched controls were less likely to fully breastfeed at 4 months, compared with normal-BMI controls. (4) Conclusion: SA is delayed in women with type 2 diabetes when compared to BMI-matched and normal-BMI women. Women with type 2 diabetes are less likely to fully breastfeed, at hospital discharge and by 4 months postpartum, compared to women with normal-BMI.

## 1. Introduction 

Lactation has significant long-term metabolic benefits for women [1]; however, few papers have assessed breastfeeding rates in women with type 2 diabetes. Two small studies suggested predominant breastfeeding beyond hospital discharge is half as frequent in women with type 2 diabetes than in women with type 1 diabetes [2] or gestational diabetes [3]. One study comparing women with type 2 diabetes to women with gestational diabetes and non-diabetic women was inconclusive [4].

Reduced breastfeeding rates may be particularly important in this population. Maternal type 2 diabetes, and co-morbid obesity present in pregnancy results in foetal programming that may contribute substantially to obesity, diabetes, and metabolic syndrome in offspring. Importantly, these effects may be ameliorated by breastfeeding [5].

Secretory activation (SA) is the onset of copious milk production, which typically commences 24–72 h postpartum [6,7]. The precise physiology underlying SA is complex, although complete placental removal (resulting in a rapid drop in progesterone) is essential [8]. SA more than 72 h postpartum is considered delayed and is associated with reduced breastfeeding [9]. Delayed SA has been postulated as one possible reason for lower breastfeeding rates in women with type 2 diabetes. Many factors are known to contribute to delayed SA including Caesarean section, early formula supplementation, maternal stress during parturition, poor infant latching, suboptimal breastfeeding, and infant prematurity [10], many of which are more common in women with type 2 diabetes. Insulin resistance per se has also been theorised as a potential mechanism for delayed SA, given the integral role of insulin in milk production [11]. However, no previous studies have examined SA in women with type 2 diabetes.

The gold standard for measuring SA is repeated weighing of the infant before and after each feed to determine when there is a sudden increase in milk volume [12,13]. However, this method is unfeasible, particularly with early postpartum discharge. More commonly, maternal sensation of milk ‘coming in’ is used as a surrogate for SA (sensitivity of 71% and specificity of 79% compared to test weighing) [12]. Alternatively, SA onset can be measured by changes in milk components indicative of lactocyte tight junction closure and milk synthesis, including sodium, citrate, lactose, fat, and protein [14]. Five studies (summarised in Polinez [15]) have validated citrate, lactose, protein, and sodium as markers of SA, with good correlation between these biomarkers and milk production. For example, milk production rapidly increases until approximately 96 h postpartum, thereafter increasing more gradually over several weeks to final maximal milk volume [16]; in healthy multiparous women, the end of this initial rapid increase (the inflexion point) corresponds to mean citrate of 3.6 mM [14]. In human lactation, citrate is recognised as a reliable marker of SA [17]. Total milk fat also increases with SA; however, change in fat concentration may be less consistent as a marker of SA because breastmilk fat content varies according to milk volume stored within the breast and throughout a breastfeeding episode [14]. In the single study assessing SA in women with type 1 diabetes, a slower increase in citrate and lactose was associated with delayed SA [17]. Similarly a slower increase in lactose has been observed in women with gestational diabetes [18].

The aim of this study was to examine whether SA timing differs between women with and without type 2 diabetes by analysing milk constituents. Secondary aims included exploring peripartum factors associated with SA timing; and the relationship between SA timing and breastfeeding at 4 months postpartum.

## 2. Materials and Methods

This study was conducted with approval of the Royal Brisbane and Women’s Hospital Ethics Committee (HREC/13QRBW/356). All participants gave written consent.

Participants were recruited between 15 and 40 weeks of pregnancy. Women with type 2 diabetes were recruited from a physician-led diabetes in pregnancy. Two control groups were recruited from midwife- and obstetrician-led pregnancy clinics at the same institution, after exclusion of gestational diabetes: age-, body mass index (BMI)-, and parity-matched nondiabetic pregnant women (within 5 years of age and 5 BMI points); and age-and parity-matched nondiabetic pregnant women with normal BMI (18.5 to 25 kg/m^2^). 

Inclusion criteria were women ≥18 years, regardless of other pre-existing conditions or gestational disorders. Exclusion criteria were women unable to communicate sufficiently in English or unable to give informed consent, intention not to breastfeed, history of prolactinoma, and gestational or type one diabetes.

### 2.1. Data and Sample Collection

Clinical data was collected for all participants including:

Baseline data (collected after 28 weeks of gestation and prior to delivery) in person and from medical notes:

Sociodemographic details, medical history, medication use, breastfeeding intentions (via a validated survey [11]), and previous breastfeeding experience. 

### 2.2. Post-Delivery Data (Collected from Hospital Medical Records)

Third trimester HBA1c of women with T2DM, predelivery insulin dose, gestation at birth, birth type, maternal and neonatal birth complications, and infant feeding methods.

### 2.3. Postpartum Data (Collected by Telephone 4 Months Postpartum)

Infant feeding method (fully breastfed, non-fully breastfed, entirely formula fed), and reasons for formula introduction or breastfeeding cessation if applicable. 

### 2.4. Breastmilk Collection and Breastmilk Analysis

Women were asked to collect up to 2 mL of breastmilk from 12–24 h postpartum and thence approximately every 12 h for five days (total of ten samples). Samples were collected by hand expression from the left breast prior to breastfeeding. Both hospital and home samples were kept frozen and thawed only at point of analysis.

Breastmilk fat content was determined by a modified creamatocrit method [19]. Breastmilk total protein concentration was determined using the assay described by Mitoulas [20] and a human milk protein standard derived using the Kjeldhal method [21]. Citrate and lactose were analysed by enzymatic methods [17,20]. 

### 2.5. Assay Validation and Quality Control 

Methodology was validated on non-study human breastmilk samples as follows: The creamatocrit method for fat content has been previously validated against the gravimetric method [22]. The recovery of a known amount of protein added to the milk samples was 101% ± 2.1% (*n* = 6). The detection limit of this assay was 0.037 g/L and the inter-assay coefficient of variation (CV) was 15% (*n* = 10). Recovery of a known amount of lactose added to the milk samples was 99.3% ± 1.6% (*n* = 6), with assay detection limit of 12 g/L and inter-assay CV of 10% (*n* = 9). Citrate concentration was similarly determined, and recovery of a known amount of citrate added to the milk samples was 101% ± 3.5% (*n* = 6), with an assay detection limit of 0.001 g/L and inter-assay CV of 13% (*n* = 8). 

### 2.6. Statistical Methods

Recruitment numbers allowed for high dropout (20%) and difficulty with sample collection, such that analyses would be robust for 10 subjects per group, and able to detect a 20% difference in SA timing with 90% power. Calculations were based on a study of SA in women with type 1 diabetes [17]. 

Comparisons of baseline demographic data and other parameters between groups were performed using one-way ANOVA for continuous variables, using the Shapiro–Wilk test to confirm normality, and Tukey’s multiple comparisons test to assess significance. Categorical variables were assessed using Fisher’s exact test. Analyses were performed using GraphPad Prism version 9.0 (www.graphpad.com, (accessed on 3 February 2022)) for Macbook, GraphPad Software, San Diego, California USA.

Breastmilk analyses were undertaken using R package sicegar version 0.2.3 (https://cran.r-project.org/package=sicegar, (accessed on 3 February 2022)) [23]. Mean citrate, lactose, protein, and fat concentrations were plotted for women in each group in 12-hour intervals from birth onwards; polynomial curve fitting was used to assess differences between groups.

Further analysis of citrate concentration changes over time was performed. Samples with data from fewer than three time points or not reaching a plateau concentration were excluded. A three-parameter sigmoidal model was fitted, as previously described [23]. Time to the midpoint of the rapid citrate rise, citrate concentration at the midpoint, and time to reach plateau citrate concentration were calculated for each woman, with values compared between the three groups. Time to the start of the citrate concentration plateau was also compared to the perceived time of milk ‘coming in’ for each woman providing this information. Outliers were identified visually and confirmed using the robust regression and outlier removal (ROUT) [24] method using Q of 10%. Time to reach specific citrate concentrations as previously reported in multiparous women for specific daily breastmilk volumes was also measured (i.e., mean citrate 2.4 millimolar (mM) which corresponds to mean milk production of 350 mL/day; and 3.6 mM which corresponds to 560 mls/day—mean peak volume) [14,25]. Mean citrate concentrations in each group were compared between women with type 2 diabetes and each control population at several timepoints, using one-way ANOVA. The proportion of women in each group whose breastmilk had reached a citrate concentration 3.6 mM, assessed within 12 hourly intervals postpartum, was also determined. Analyses of other variables associated with SA was performed at the timepoint where a significant difference in SA was seen between groups. Groups were compared using Fisher’s exact test. 

Principal component analysis (PCA) was performed, considering milk concentrations of fat, protein, lactose, and protein at 72 h postpartum, hospital formula use, feed method at discharge, and infant hypoglycaemia as variables, within the three groups. 

For women with type 2 diabetes, third trimester HbA1C was also considered, and correlation analysis used to examine the association between pre-delivery insulin dose per kilogram and time to reach citrate thresholds defined above.

## 3. Results

### 3.1. Cohort

Eighteen women per group (type 2 diabetes, BMI-matched controls, and normal-BMI controls) were recruited. Demographics were similar between the groups, including intention to breastfeed (Supplementary Table S1). Fourteen women with type 2 diabetes, 10 BMI-matched controls, and 12 normal-BMI controls provided enough breastmilk samples for specimen analysis: all subsequent results are based on these data. Dropout was not significantly different between groups (*p*-value = 0.6). Baseline demographics were similar in these analysed women (Table 1), with the exception that women with type 2 diabetes were more likely to be of Southeast Asian origin than BMI-matched controls. In women with type 2 diabetes, mean HBA1c was 5.5% (standard deviation ± 0.4) (Supplementary Table S2).

### 3.2. Clinical Parameters

Higher rates of neonatal hypoglycaemia were noted in babies of women with type 2 diabetes (10 of 14 babies, (71%)) compared to both control groups (0 of 10, and 1 of 12 (8%) babies from BMI-matched and normal-BMI women, respectively). Sixteen babies received supplemental feeds with formula before discharge, for various reasons (Supplementary Table S3). Nine of the 15 (60%) infants in whom time of first formula supplementation was recorded commenced formula within 24 h postpartum, before SA was possible [7]. Six of nine (67%) who received formula in the first 24 h postpartum were infants of women with type 2 diabetes.

Rates of formula supplementation in hospital were higher in the infants of women with type 2 diabetes compared to women with normal-BMI (*p*-value = 0.049), with a trend in the BMI-matched group (*p*-value = 0.09).

At discharge, women with type 2 diabetes were less likely to be fully breastfeeding, compared with either control group (Table 2). By 4 months postpartum, of the 26 women fully breastfeeding at discharge, 13 (50%) were still fully breastfeeding. Conversely, none of the seven women not fully breastfeeding at discharge were fully breastfeeding at 4 months postpartum. Women with type 2 diabetes were less likely to be fully breastfeeding at 4 months postpartum compared with the normal-BMI group; however, there was no difference in full breastfeeding at 4 months between women with type 2 diabetes and the BMI-matched group. Data for three groups combined found elevated BMI above 25 and 30, respectively, was significantly associated with not breastfeeding at 4 months postpartum (Supplementary Table S4).

### 3.3. Breastmilk Analysis

**Figure 1:**
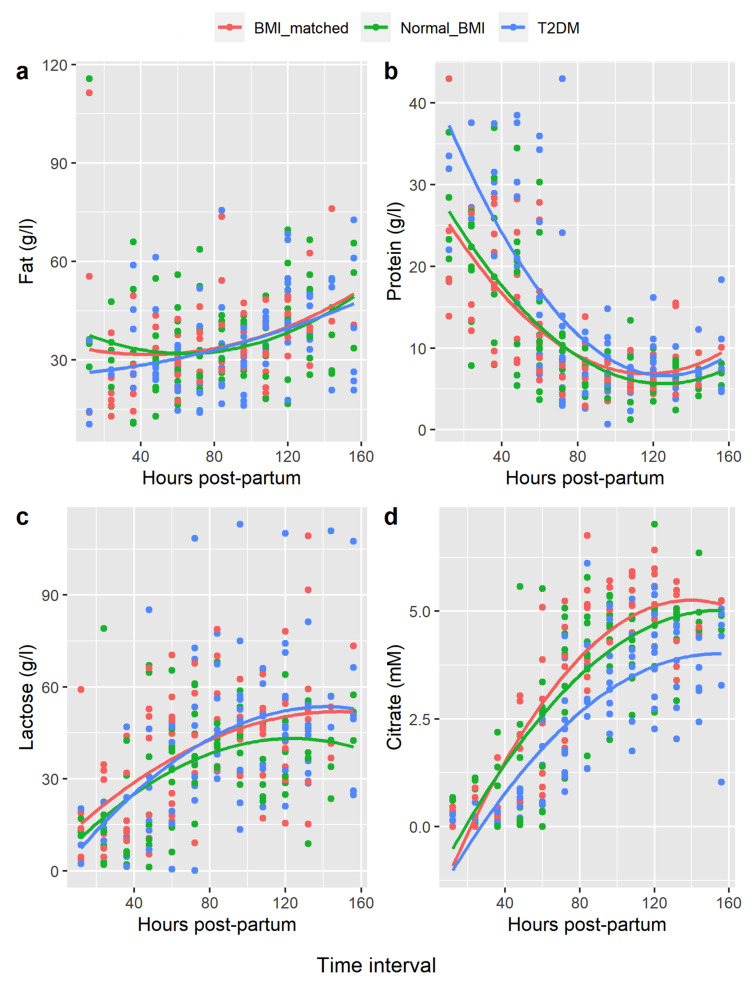
Polynomial Regression of Breastmilk Constituents Hours Postpartum. Legend: (**a**) = fat, (**b**) = protein, (**c**) = lactose, (**d**) = citrate. *x*-axis = hours postpartum, mean values for each woman compared at 12 hourly intervals. *y*-axis = concentration breastmilk constituent in gram/litre (g/L) or millimolar (mM).

Polynomial curve fitting showed the changes in concentration of fat, protein, and lactose over time postpartum in each group were similar. However, citrate rose more quickly in both the BMI-matched and normal-BMI groups compared with the type 2 diabetes group (BMI-matched vs. type 2 diabetes, *p*-value = 0.001; normal-BMI vs. type 2 diabetes, *p*-value < 0.001 (Figure 1).

Citrate plotted over time for each woman showed a sigmoidal pattern in all women (i.e., low initial levels, rapid rise, then an inflexion point, followed by a more gradual rise to final plateau concentrations, consistent with completion of SA). Pre-SA citrate concentrations were not significantly different between groups. However, mean citrate midpoint concentrations were lower in type 2 diabetes women, compared with both controls. The mean citrate at the post-rise plateau was also lower in the type 2 diabetes group compared to both control groups (Table 3). Times to reach these thresholds were not significantly different between groups; however, the numbers in this analysis were small due to incomplete collections and/or insufficient volume for analysis. 

Estimated time of SA (obtained from women estimating when their milk ‘came in’) was available for nineteen women: fifteen of these women also had data for time of commencement of citrate plateau. After removal of two outliers, time of perceived SA correlated well with time of commencement of citrate plateau, (r^2^ = 0.71, *p*-value < 0.001).

Considering when breastmilk citrate concentration reached 3.6 mM (i.e., the value previously associated with a plateau of copious milk production in non-diabetic women [14]), a relationship suggesting fewer women with type 2 diabetes reached this threshold compared to both control groups was initially evident at 72 h and persisted until 108 h, which was significant at 84 h postpartum (i.e., 12 h after usual time for SA) ((*p*-value = 0.002 when with BMI-matched controls; or (*p*-value = 0.009 when compared with normal-BMI controls) (Supplementary Table S5 and Figure S1). Based on this finding, further analyses of other variables associated with SA compared women reaching the threshold of 3.6 mM by 84 h with those who had not. Infant hypoglycaemia was associated with failure to reach this threshold. Failure to reach this threshold was not associated with rates of full or any breastfeeding at discharge or 4 months postpartum (Supplementary Table S6). 

**Figure 2:**
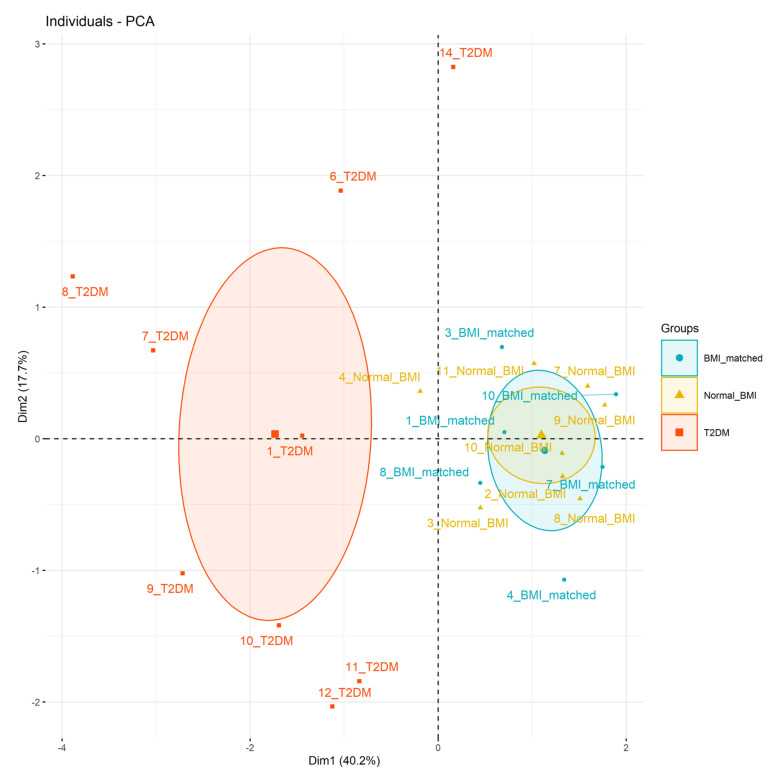
Principal component analysis. Data considered: principal component 1 (*x*-axis, 40% variance) infant hypoglycaemia, (correlation 0.78), formula use in hospital (correlation 0.75), citrate concentration at 72 h (correlation 0.74), principal component 2 (*y*-axis, 18% variance) protein concentration at 72 h (correlation 0.54), and lactose concentration at 72 h (correlation 0.51).

Principal components analysis (Figure 2) showed the type 2 diabetes group clustered separately from the BMI-matched and normal-BMI groups (Figure 2). Principal components 1, 2, and 3 had eigenvalues >1, accounting for 40%, 18%, and 16% of overall variance, respectively. The main factors in each principal component were 1: infant hypoglycaemia, hospital formula use, and citrate concentration at 72 h; 2: protein and lactose concentrations at 72 h; and 3: fat content at 72 h (Supplementary Table S7).

Association between glycemia, insulin dose, and citrate was examined. In the fourteen women with type 2 diabetes, there was no association between insulin dose (in units/kg) and pre-delivery BMI (*p*-value = 0.18) or HBA1c (*p*-value = 0.94). Excluding data for two women not taking metformin (an insulin sensitiser), insulin dose correlated positively with time to reach the midpoint of rapid citrate rise (r^2^ = 0.72, *p*-value = 0.008, *n* = 8), and with time to commencement of citrate plateau (r^2^ = 0.84, *p*-value = 0.001, *n* = 8), as well as time to citrate concentrations of 2.4 mM (r^2^ = 0.94, *p* < =0.001, *n* = 8) and 3.6 mM (r^2^ = 0.83, *p*-value = 0.01, *n* = 6).

## 4. Discussion

To our knowledge our pilot study is the first to examine postpartum breastmilk constituents and factors affecting breastfeeding in women with type 2 diabetes. We have demonstrated that citrate concentrations rose more slowly, and plateaued at a lower concentration, in women with type 2 diabetes compared to BMI-matched and normal-BMI controls, supporting our hypothesis that SA timing may be delayed in women with type 2 diabetes. Moreover, women requiring larger insulin doses to achieve glycaemic control during pregnancy (a surrogate measure for insulin resistance) had later SA. However, there were also significant differences in clinical parameters between women with type 2 diabetes and the control groups possibly contributing to altered SA, including higher rates of infant hypoglycaemia, earlier formula use, and lower rates of full breastfeeding at discharge in women with type 2 diabetes.

### 4.1. Role of Citrate in Lactation and as a Marker of SA 

Citrate profiles differed significantly in women with type 2 diabetes compared to both control groups. Absence of differences in total protein, lactose, and fat between groups suggests that tight junction closure may not differ between these groups; rather, the delay in citrate increase may relate to delay in milk synthesis. Citrate is synthesised from acetate and glucose; and the breast requires glucose to support milk secretion [26]. The delay in breastmilk citrate secretion in women with type 2 diabetes may reflect a delay in glucose and oxygen uptake, compared to the other women. 

Although many women in this study could not provide a time for perceived SA, in those who could there was strong correlation between time of perceived SA and time of citrate plateau, a proxy indicator of volume. This confirms that the previously noted value of maternal perception of SA in women without diabetes [12] and with gestational diabetes [27] is also an indicator for women with type 2 diabetes.

### 4.2. Breastmilk Constituents in Obesity and Type 2 Diabetes

Another potential explanation for different concentrations of breastmilk constituents between groups is metabolic differences and differing maternal diet. For example, women with obesity may have a lower intake of n-3 and n-6 polyunsaturated fatty acid in their diet, and also lower breastmilk concentrations compared to normal weight women [28]. Future research examining breastmilk constituents in full lactation, including free fatty acid analysis, and a comprehensive assessment of the role of diet in determining breastmilk constituents would help to differentiate this. 

### 4.3. Insulin Resistance and SA in Women with Type 2 Diabetes

In women with type 2 diabetes, larger insulin dose at delivery (in units/kg) associated with longer time for citrate rise, and, by inference, later SA. Third trimester HBA1cs were similar between participants, with glycaemic control reaching the target of <6% [29] in all women except one (Supplementary Table S2); thus, the required insulin dose can be considered a proxy for insulin resistance. Our results therefore suggest women with greater insulin resistance may have later SA and may produce less milk initially. The importance of insulin receptor expression in mammary differentiation, milk lipid, protein, and lactose synthesis has been shown definitively in the mouse model [30,31]; however, in humans the role of insulin and insulin resistance on breast development and milk production remains understudied [11]. A study of women with polycystic ovarian syndrome showed decreased breast growth in pregnancy was associated with decreased exclusive and any breastfeeding, being more common in older women, and women with higher first trimester BMI, and higher fasting insulin concentrations in this patient group [32]. Another study showed ongoing impaired glucose tolerance postpartum (a harbinger of type 2 diabetes) is associated with decreased predominant breastfeeding to 12 months (32). Hummel [33] found decreased duration of breastfeeding in gestational diabetes was independently associated with insulin treatment (versus diet manipulation). Our study did not use a formal insulin clamp so any relationship between insulin resistance and SA timing can only be inferred; however, our data suggest a potential role for insulin, and insulin resistance, in determination of lactation success.

### 4.4. Obesity and SA

Given the high prevalence of elevated BMI in pregnant women with type 2 diabetes [34], we recruited a BMI-matched control group without type 2 or gestational diabetes to consider the effect of BMI per se on SA. No difference in citrate profiles between BMI-matched and normal-BMI control groups was found, suggesting that obesity/overweight per se did not affect SA. Infants of women with type 2 diabetes were less likely to be fully breastfed at hospital discharge than BMI-matched and normal-BMI controls; there was no difference between the control groups yet similarly lower rates of full breastfeeding were seen at 4 months postpartum in both type 2 diabetes and BMI-matched groups, compared with women with normal-BMI. Obesity is associated with delayed breastfeeding initiation, delayed SA assessed by maternal perception, and decreased full/any breastfeeding, compared to women with normal BMI [6,35], attributed to anatomical, psychological, sociocultural, and physiological factors [36]. Physiological factors have been shown in animal models, with decreased milk production in obese cows [37] and obese rats [38]; however, these studies did not explore insulin resistance. As BMI correlates with both type 2 diabetes and gestational diabetes, our use of a high BMI non-diabetic control group may have selected a relatively non-insulin resistant obese/overweight population. Lack of delayed SA in this control group suggests insulin resistance may be more important in determining SA than obesity. That there was no difference between the type 2 diabetes and BMI-matched groups in full breastfeeding rate at 4 months suggests factors other than delayed SA contribute towards establishing full breastfeeding in women with obesity in the medium term.

### 4.5. Formula Supplementation, Infant Hypoglycaemia, and SA

Our study showed women with type 2 diabetes were more likely to have infants with hypoglycaemia than BMI-matched and normal-BMI controls. Perhaps unsurprisingly, infants of type 2 diabetic mothers were more likely to be supplemented with formula in hospital than normal-BMI controls, with a similar trend when compared to the BMI-matched group. PCA showed formula supplementation in hospital and hypoglycaemia were both important factors separating women with type 2 diabetes from BMI-matched and normal-BMI groups, along with citrate concentration at 72 h. Our study did not show a clear association between formula use and SA but our study (powered to assess SA through citrate concentration assessment) is likely underpowered for this outcome. Sixty percent of formula feeds commenced within 24 h postpartum, mostly to meet hospital formula-fed quotas (30 mls/kg/day prior to 24 h of age) in babies with medical illness (here, predominantly hypoglycaemia). Whilst these mothers reported low supply of breastmilk, the physiological production of breastmilk during this time is approximately 60 mls over 24 h [16]. Thus, the prescribed formula dosing far exceeds maximal possible physiological milk production within this timeframe and likely exceeds physiological requirements. In some [39,40,41] but not all [42] studies, early formula introduction is associated with poorer breastfeeding outcomes. We acknowledge that early formula supplementation may contribute to slower citrate rise, via less infant suckling of the maternal breast [6]. A recent trial that randomised women to breastfeeding alone or breastfeeding with additional expressing supports this theory: increased breastmilk citrate correlated with larger volumes of expressed breastmilk in women expressing and breastfeeding regularly early postpartum, compared to women who breastfed only [43]. Alternatively, it may be that formula is more likely to be given to infants of women who perceive poor supply in the setting of impaired SA [39].

Our study also showed an association between lower citrate concentration at 84 h and neonatal hypoglycaemia, noting that most hypoglycaemia occurred in infants of women with type 2 diabetes. Untreated hypoglycaemia is associated with long-term cognitive deficits [44]; it is unknown whether acute hypoglycaemia itself interferes with the infant’s ability to breastfeed. The number of breastfeeds in the first 24 h is associated with breastfeeding at 4 months in type 2 diabetes [2], but whether the number of breastfeeds is associated with prior infant hypoglycaemia is unclear. A recent study showed giving infants with early hypoglycaemia donor milk rather than formula is associated with increased full breastfeeding at 6 months postpartum [45]. Further research could assess whether managing hypoglycaemia with non-enteral methods and/or donor milk may affect SA and longer-term breastfeeding.

### 4.6. Demographic and Iatrogenic Factors Previously Demonstrated to Affect SA

Educational status, socioeconomic status, and breastfeeding intentions were similar between our three groups, as was mode of delivery, regional anaesthesia, and nursery admission (Table 1). This suggests that these factors were not responsible for differences in citrate concentrations between groups.

### 4.7. Strengths and Limitations

Our pilot study has several strengths. We expected, and powered our study, for dropout, anticipating difficulty with colostrum and breastmilk collection for women with neonates. Even after dropout, groups were matched for many variables known to affect SA, including BMI, age, and parity. Our use of both BMI-matched and normal-BMI controls allowed exploration of the separate effects of type 2 diabetes and obesity upon breastfeeding. The rate of full breastfeeding at 4 months in normal BMI controls (67%) was similar to that reported by previous studies in Australian women (64%) [46], suggesting this control sample is representative of the population. 

However, we also acknowledge small sample size. As expected, many women had difficulty with sample collection, resulting in missing data points. We did not have objective data relating to breastmilk volume, making it difficult to differentiate whether low citrate concentrations reflected metabolic abnormalities related to diabetes or were a marker of impaired/delayed SA (or all three). Further, we did not have data on women’s diet, which may influence breastmilk composition. Whilst laboratory analysis of milk was blinded to the groups, collection of data at 4 months postpartum was not. Differences in clinical parameters between women with type 2 diabetes and our control groups (saliently, infant hypoglycaemia, and formula supplementation) mean our study remains hypothesis-generating with respect to whether type 2 diabetes independently affects SA. None-the-less, our pilot study provides sufficient evidence to justify a larger study powered to explore contributory factors to poorer breastfeeding rates in women with type 2 diabetes, and points towards future interventional trials addressing this important issue.

## 5. Conclusions

In conclusion, SA was delayed in women with type 2 diabetes compared with both BMI-matched and normal-BMI controls. However, delayed SA per se was not associated with reduced full or any breastfeeding at 4 months postpartum. Larger studies are required to confirm whether peripartum events, including infant hypoglycaemia and early formula introduction, contribute to slower rise in citrate in women with type 2 diabetes, and whether delayed SA translates into poorer long-term breastfeeding outcomes in women with type 2 diabetes.

## Figures and Tables

**Table 1:** Pre-Delivery Data: Women Who Provided Breastmilk For Analysis.

	T2DM (a)	BMI-Matched (b)	Normal BMI (c)	Comparison, *p*-Value
*n*	14	10	12	
Pre-pregnancy BMI	30.6 ± 6.1	27.9 ± 4.7	21.1 ± 1.6	<0.001 *** a vs. b, 0.37 a vs. c, <0.001 *** b vs. c, 0.005 **
Pre-delivery BMI	33.7 ± 6.8	33.1 ± 5.5	25.8 ± 2.6	0.001 ** a vs. b, 0.96 a vs. c, 0.002 ** b vs. c, 0.009 **
Age at due date (years)	35.1 ± 5.2	33.1 ± 5.6	34.0 ± 4.1	a vs. b, vs. c, 0.63
Education (degree or higher)	(11/14) 79%	(5/10) 50%	(8/12) 67%	a vs. b, 0.20 a vs. c, 0.67
Parity (1 or greater)	(10/14) 72%	50% (5/10)	75% (9/12)	a vs. b, 0.40 a vs. c, >0.99
Breastfeeding intentions score (/16)	14 ± 2	15 ± 2	15 ± 1	a vs. b vs. c, 0.31
Ethnicity (Caucasian vs. SE Asian)	7/14 (50%)	10/10 (100%)	10/12 (83%)	a vs. b, 0.02 * a vs. c, 0.11
Income >$AUD 50,000 AUD (>poverty threshold)	(11/14) (79%)	(10/10) 100%	10/10 (100%)	a vs. b, 0.24 a vs. c, 0.24
Income >$AUD 100,000 (>median income)	4/14 (29%)	5/10 (50%)	5/10 (50%)	a vs. b, 0.40 a vs. c, 0.40
Hypertension	2/14	0/10	0/12	a vs. b, 0.49 a vs. c, 0.48
Polycystic ovarian syndrome	2/14 (14%)	2/10 (20%)	0/12 (0%)	a vs. b, >0.99 a vs. c, 0.48
Asthma	4/14 (29%)	3/10 (30%)	1/12 (8%)	a vs. b, >0.99 a vs. c, 0.36
Hypothyroidism	3/14 (21%)	1/10 (10%)	1/12 (8%)	a vs. b, 0.61 a vs. c, 0.60
Depression	2/14 (14%)	1/10 (10%)	2/12 (17%)	a vs. b, >0.99 a vs. c, >0.99
Other major systemic medical condition	1/14 (7%)	0/10 (0%)	1/12 (0%)	a vs. b, >0.99 a vs. c, >0.99

Legend: BMI = Body mass index (kilogram/meter ^2^); SE = Southeast; AUD = Australian dollars; T2DM = Type 2 diabetes; Groups: a = T2DM, b = BMI-matched, c = normal-BMI, * *p*-value ≤ 0.05; ** *p*-value ≤ 0.01, *** *p*-value ≤ 0.001.

**Table 2:** Post-Delivery Data: Women Who Provided Breastmilk for Analysis.

	T2DM (a)	BMI-Matched (b)	Normal BMI (c)	Comparison, *p*-Value
	14	10	12	
Infant birthweight (g)	3248 ± 354	3367 ± 339	3316 ±469	a vs. b vs. c, 0.76
Gestational age at delivery (weeks)	38.7 ± 1.1	39.4 ± 1.6	39.2 ± 0.9	a vs. b vs. c, 0.39
Vaginal birth (vs. caesarean section)	7/14 (50%)	7/10 (70%)	6/12 (50%)	a vs. b, 0.42 a vs. c, >0.99
Blood loss greater than 500 mls	3/14 (21%)	4/10 (40%)	3/12 (25%)	a vs. b, 0.39 a vs. c, 0.99
Pre-ecclampsia	2/14 (14.2%)	0/10 (0%)	0/10 (0%)	a vs. b, 0.49 a vs. c, 0.48
Regional anaesthesia for delivery	10/14 (71%)	8/10 (80%)	9/12 (75%)	a vs. b, >0.99 a vs. c, >0.99
SCN or ICN admission	4/14 (29%)	1/10 (10%)	2/12 (17%)	a vs. b, 0.36 a vs. c, 0.65
Neonatal hypoglycaemia	10/14 (71%)	0/10 (0%)	1/12 (8%)	a vs. b, >0.001 *** a vs. c, 0.002 **
Breastfed in first hour of life	13/14 (92.86%)	7/10 (70.0%)	11/12 (92%)	a vs. b, 0.27 a vs. c, >0.99
Supplemented formula during admission	10/14 (71%)	3/10 (30%)	3/12 (25%)	a vs. b, 0.09 a vs. c, 0.047 * b vs. c, >0.99
Breastfeeding fully ^+^ discharge (vs. breastmilk and formula)	6/14 (43%)	9/10 (90%)	11/12 (92%)	a vs. b, 0.03 * a vs. c, 0.01 * b vs. c, >0.99
Breastfeeding fully ^+^ at 4 months postpartum	3/13 (23%)	2/9 (22%)	8/12 (67%)	a vs. b, >0.99 a vs. c, 0.047 * b vs. c, 0.08
Any breastfeeding at 4 months postpartum	8/13 (62%)	5/9 (56%)	10/12 (83%)	a vs. b, >0.99 a vs. c, 0.38 b vs. c, 0.33

+ Note: ‘fully’ refers to only breastmilk given without other liquids or solids with the exception of vitamins, minerals, or medicines at the time of survey and for the preceding 24 h. Infant formula may have been previously given. Legend: BMI = Body mass index (kg/m^2^); T2DM = Type 2 diabetes; SCN = Special care nursery; ICN = Intensive care nursery; Groups: a = T2DM, b = BMI-matched, c = normal-BMI, * *p*-value ≤ 0.05; ** *p*-value ≤ 0.01; *** *p*-value ≤ 0.001.

**Table 3:** Breastmilk Citrate Concentrations Postpartum.

Variable	T2DM (Group a)	BMI-Matched (Group b)	Normal BMI (Group c)	Comparison, *p*-Value
Plateau Citrate Value for all available curves (mM) (*n* = 30)	*n* = 10 3.63 ± 0.60	*n* = 10 4.59 ± 0.66	*n* = 10 4.33 ± 0.60	0.005 ** a vs. b, 0.005** a vs. c, 0.04* b vs. c, 0.64
Time to Plateau Citrate Value for all available curves (*n* = 30)	*n* = 10 82.25 ± 22.40	*n* = 10 76.25 ± 12.29	*n* = 10 67.69 ± 19.21	a vs. b vs. c, 0.23
Mean citrate at midpoint of rapid rise (mM)	*n* = 10 2.06 ± 0.38	*n* = 10 2.60 ± 0.38	*n* = 10 2.46 ± 0.34	0.005 ** a vs. b, 0.005** a vs. c, 0.04* b vs. c, 0.64
Time to mean citrate at midpoint of rapid rise	*n* = 10 68.7 ± 12.33	*n* = 10 59.86 ± 12.69	*n* = 10 55.09 ± 17.72	a vs. b vs. c, 0.12
Time to Citrate 2.4 (mM)	*n* = 10 72.92 ± 16.20	*n* = 10 58.08 ± 13.33	*n* = 10 55.19 ± 16.87	a vs. b vs. c, 0.04* a vs. b, 0.10 a vs. c, 0.04* b vs. c, 0.91
Time to Citrate 3.6 (mM)	*n* = 7 77.88 ± 21.38	*n* = 10 65.83 ± 11.90	*n* = 10 63.14 ± 17.39	a vs. b vs. c, 0.20

Legend: T2DM = Type 2 diabetes; BMI = Body mass index (kg/m^2^)**;** mM = millimolar, * *p*-value ≤ 0.05; ** *p*-value ≤ 0.01; *** *p*-value ≤ 0.001.

## Data Availability

Important data presented in this study are available in the Supplementary Materials. Further data available from the corresponding author on request and are not publicly available for privacy reasons.

## Supplementary Materials

The following supporting information can be downloaded at: https://www.mdpi.com/article/10.3390/nu14071323/s1, Table S1: Baseline Demographics of All Recruited Women, Table S2: HBA1c Women Type 2 Diabetes, Table S3: Timing/Reasons for Supplementation with Formula in Hospital, Table S4: Factors Predicting Breastfeeding at Four Months Postpartum. Table S5: Time to Reach Citrate Concentration of 3.6 mM, Figure S1: Percentage of Each Group Reaching Citrate of 3.6 mM vs. Hours Postpartum, Table S6: Variables and Association with Reaching Citrate 3.6 mM by 84 h.
