# Supplementary material for: Is Secretory Activation Delayed in Women with Type Two Diabetes? A Pilot Study
Source: Nutrients. 2022 Mar 22;14(7):1323. doi: 10.3390/nu14071323 (PMC9002373; doi:10.3390/nu14071323)
Supplement: Supplementary file 1 [file nutrients-14-01323-s001.zip › nutrients-1605231-supplementary.pdf]

Supplementary Materials

Contents

1. Table S1: Baseline Demographics of All Recruited Women

   Page 2

2. Table S2: HBA1c Women Type 2 Diabetes.

   Page 2

3. Table S3: Timing/Reasons for Supplementation with Formula in Hospital

   Page 3

4. Table S4: Factors Predicting Breastfeeding at Four Months Post-partum

   Page 4

5. Table S5: Time to Reach Citrate Concentration of 3.6 mM

   Page 5

6. Figure S1: Percentage of Each Group Reaching Citrate of 3.6mM vs Hours Postpartum

   Page 5

7. Table S6: Variables and Association with Reaching Citrate 3.6mM by 84 hours

   Page 6

Table S1. Baseline Demographics of All Recruited Women.

Variable	T2DM	BMI-matched Controls	Normal BMI Controls	Comparison, P-value
Number	18	18	18	
Pre-pregnancy BMI	31.2 +/- 6.5	29.5 +/- 5.9	21.6 +/1 2.0	a *vs.* b *vs.* c, <0.001***<br>a *vs.* b, 0.37<br>a *vs.* c, <0.001***<br>b *vs.* c, 0.005
Pre-delivery BMI	33.7 +/- 6.8	32.4 +/- 5.9	25.8 +/- 2.5	a *vs.* b *vs.* c, 0.002**<br>a *vs.* b, 0.84<br>a *vs.* c, 0.002**<br>b *vs.* c, 0.02*
Age at due date (years)	34.8 +/- 5.1	32.7 +/- 4.7	34.1 +/-4.1	a *vs.* b *vs.* c, 0.35
Education (Tertiary education)	66% (13/19)	56% (10/18)	73% (13/18)	a *vs.* b, 0.51<br>a *vs.* c, >0.99
Breastfeeding Intentions Score (/16)	14 +/- 2	14 +/- 3	15 +/- 1	a *vs.* b *vs.* c, 0.54

Compared using one way ANOVA

Legend: BMI=Body mass index (kilogram/meter$^{2)}$
Groups: a=T2DM, b=BMI-matched, c=normal BMI

Table S2: HBA1c (mmol/mol) Women Type 2 Diabetes

**6.3**	**5.1**	**5.1**	**5.5**	**5.8**	**5.3**	**5.9**	**5.6**	**5.6**	**5.4**	**5.0**	**5.2**	**5.4**	**5.9**
Mean 5.5% +/- 0.4													

Table S3: Timing/Reasons for Initial Supplementation with Formula in Hospital

Group	Time	Reason (chart)	Reason (woman)	Feeding method (discharge)	Feeding method (4 months)
A	47	Maternal request	Not available	BF	BF
A	Not recorded	To meet 'full quota' in setting - increased work of breathing, neonatal infection, SCN admission	Poor supply	BF	FF
A	4.5	Hypoglycaemia BGL 2.6<br>To meet 'full quota' (30mls/kg/day)	Poor supply	MF	FF
A	2.0	Hypoglycaemia BGL 2.6 mmol/l<br>To meet 60mls/kg/day q3h as 'minimal maternal supply'	Not available	MF	MF
A	15	Hypoglycaemia BGL 2.8 mmol/l	Poor supply	FF	MF
A	6.5	Hypoglycaemia BGL 2.4mmol<br>To meet 'full quota' tops ups (60mls/kg/day)	Poor supply	MF	MF
A	10.5	No reason documented	Poor supply	MF	FF
A	39.0	To meet 'quota' once suck feeds started in SCN	Insufficient weight gain	MF	FF
A	91.0	Jaundice. To meet 'half quota' + BF	Jaundice	MF	MF
A	2.0	Maternal request after glucose gel given for hypoglycaemia due to maternal concerns about supply	Insufficient weight gain	MF	MF
B	11.5	Maternal request	Poor supply	MF	FF
B	32.0	No reason documented	Poor supply	BF	FF
B	10.5	Poor feeding and lethargy with risk of sepsis. BF plus formula to meet 'full quota' (30mls/kg/day) q3h	Not available	MF	Not Available
C	41.0	NG feeds due to tachypnoea/possible infection. Formula to meet 'quota' (90ml/kg/day)	Baby admitted to SCN	BF	BF
C	49.5	No reason documented	Poor supply	BF	BF
C	7.0	Maternal request	Poor supply	MF	MF

Note: Formula prescribed according to estimated needs of a fully formula fed infant, hence 'full quota' equates to 30mls/kg/day on day one, 60mls/kg/day on day two and 90mls/kg day on day three of life.

Legend: Time= time postpartum formula first given (hours to nearest half hour)
Reason (chart)= reason documented in neonatal chart at time first formula given to the infant
Reason (woman)= Reason woman gave at postpartum survey for first formula supplement
Feeding method (discharge)= Feeding method at hospital discharge
Feeding method (4 months)= Feeding method at four months postpartum
Groups: A=T2DM, B=BMI-matched, C=normal BMI. SCN=Special Care Nursery. BF = Breastfeeding only, FF = Formula Feeding only, MF = Mixed formula and Breastfeeding

Table S4: Factors Predicting Breastfeeding at Four Months Postpartum

Variable	Fully BF at 4 months	Not fully BF at 4 months	*P*- value
**BMI >25**			
**BMI >25**	4/13 (31%)	12/21(57%)	0.17
**BMI < or equal 25**	9/13 (69%	9/21 (43%)	
**BMI >30**			
**BMI >30**	2/13 (15%)	7/21 (33%)	0.43
**BMI < or equal 30**	11/13 (85%)	14/21 (67%)	
**Fully BF at discharge**	13/13 (100%)	14/21 (67%)	0.03 *
**Not fully BF at discharge**	0/13 (0%)	7/21 (33%)	
**Formula Supplementation in Hospital**	3/13 (21%)	11/21 (52%)	0.15
**Citrate 3.6mM by 84 hours post-partum**	9/13 (69%)	13/18 (59%)	>0.99
	**Any BF at 4 months**	**Not BF at 4 months**	
**BMI >25**			
**BMI >25(n=16)**	7/22 (32%)	9/12 (75%)	
**BMI < or equal 25**	15/22 (68%)	3/12 (25%)	0.03 *
**BMI >30**			
**BMI >30**	3/9 (33%)	6/9 (67%)	0.04 *
**BMI< or equal 30**	19/25 (76%)	6/25 (24%)	
**Formula Supplementation in Hospital**	7/22 (32%)	7/12 (58%)	0.16
**Citrate 3.6 by 84 hours post-partum**	14/212 (64%)	8/10 (80%)	0.68

*All groups combined, compared using Fisher's exact test, * p-value <0.05.*

Legend: BMI: Body Mass Index, BF=breastfeeding

Table S5: Time To Reach Citrate Concentration of 3.6 mM

Hours to Reach Citrate 3.6mM	Type 2 Diabetes (a)	BMI-matched (b)	Normal BMI (c)	*Comparison, P- value*
12	0/13 (0%)	0/10 (0%)	0/11 (0%)	No difference
24	0/13 (0%)	0/10 (0%)	1/11 (9%)	a *vs.* b, >0.99<br>a *vs.* c, 0.46
36	0/13 (0%)	0/10 (0%)	1/11 (9%)	a *vs.* b, >0.99<br>a *vs.* c, 0.46
48	0/13 (0%)	0/10 (0%)	1/11 (9%)	a *vs.* b, >0.99<br>a *vs.* c, 0.46
60	2/13 (15%)	3/10 (30%)	2/11 (18%)	a *vs.* b, 0.62<br>a *vs.* c, >0.99
72	3/13 (23%)	6/10 (60%)	7/11 (64%)	a *vs.* b, 0.04 *<br>a *vs.* c, 0.09
84	4/12 (33%)	10/10 (100%)	10/11 (91%)	a *vs.* b, 0.002 **<br>a *vs.* c, 0.009 **
96	5/12 (42%)	10/10 (100%)	10/11 (91%)	a *vs.* b, 0.005 **<br>a *vs.* c, 0.03 *
108	6/12 (50%)	10/10 (100%)	10/11 (91%)	a *vs.* b, 0.01 *<br>a *vs.* c, 0.07
120	9/13 (69%)	10/10 (100%)	10/11 (91%)	a *vs.* b, 0.10<br>a *vs.* c, 0.33
132	9/13 (69%)	10/10 (100%)	10/11 (91%)	a *vs.* b, 0.01 *<br>a *vs.* c, 0.33

*Compared using Fisher's exact test, *denotes p-value≤0.05, **denotes p-value≤0.01.*

Legend: mM=millimolar, BMI=Body mass index (kilogram/meter^{2})
Type 2 Diabetes=Group a(n=13), BMI-Matched=Group b(n=10), Normal-BMI=Group c (n=11)
Denominators vary as information about some variables not available for all women

Figure S1

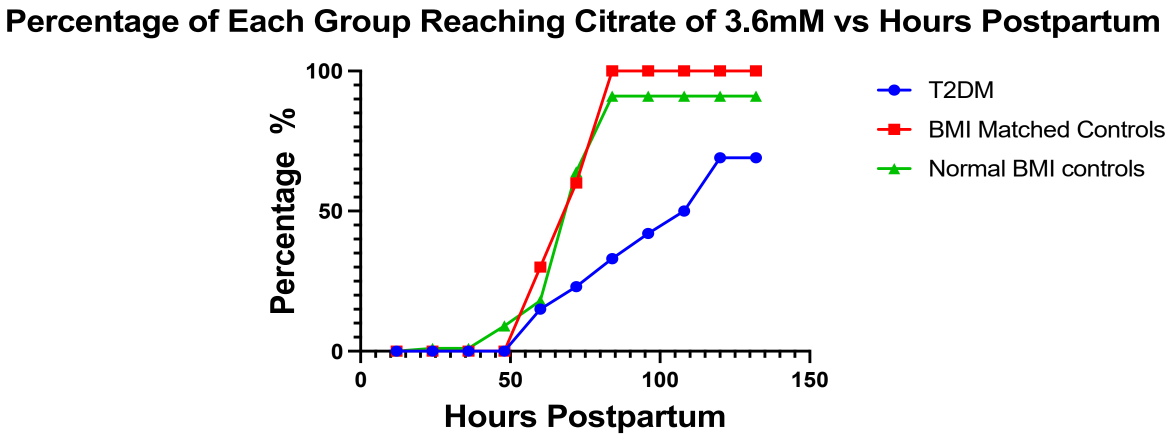

Table S6: Variables and Association with Reaching Citrate 3.6mM by 84 hours

	Citrate 3.6mM reached by 84 hours	Citrate 3.6mM not reached by 84 hours	*P- value*
**Formula use in hospital**	7/23 (30%)	6/10 (60%)	0.11
**Fully BF at hospital discharge**	20/23 (87%)	6/9 (56%)	0.33
**ICN/SCN admission**	6/18 (33%)	3/9 (33%)	0.65
**Perception SA after 72 hours**	6/13 (46%)	2/8 (25%)	>0.99
**Regional anaesthesia**	16/23 (70%)	8/10 (80%)	>0.99
**Blood loss of >500mls peripartum**	8/16 (50%)	2/10 (10%)	0.39
**Infant hypoglycaemia**	2/23 (9%)	8/11 (73%)	<0.001 ***
**Birth type vaginal**	14/23 (73%)	6/10 (60%)	>0.99
**Birthweight >3.5kg**	8/22 (73%)	3/9 (27%)	>0.99

*Compared using Fisher's exact test, ***denotes p-value≤0.001.*

Legend: BF=breastfeeding, ICN=Intensive Care Nursery, SCN=Special Care Nursery, SA=Secretory Activation, mM=millimolar.
Note: 8/9 hypoglycaemic infants were in T2DM group.
Denominators vary as information about some variables not available for all women.
